# Determinants of dietary diversity among women of reproductive age in two different agro-ecological zones of Rongai Sub-County, Nakuru, Kenya

**DOI:** 10.29219/fnr.v63.1553

**Published:** 2019-01-18

**Authors:** Maureen Wanjiru Gitagia, Rose Chepchirchir Ramkat, Dorothy M Mituki, Celine Termote, Namukolo Covic, Maureen Jepkorir Cheserek

**Affiliations:** 1 Department of Human Nutrition, Faculty of Health Science, Egerton University, Rift Valley, Kenya; 2 Department of Biological Sciences, Moi University, Eldoret, Rift Valley, Kenya; 3 Diets from Sustainable Food Systems Initiative, Bioversity International, Nairobi, Kenya; 4 International Food Policy Research Institute, Poverty Health and Nutrition Division, Agriculture for Nutrition and Health, Addis Ababa, Ethiopia

**Keywords:** Agro-biodiversity, agro-ecological zones, women’s dietary diversity, determinants, rural Kenya

## Abstract

**Background:**

Empirical evidence on the link between agrobiodiversity and dietary diversity appears to be inconclusive. Thus, there arises a need to determine other factors that could significantly influence dietary diversity in different agro-ecological zones, as factors may vary from region to region.

**Objective:**

The objective of this study was to document the status of agrobiodiversity and dietary diversity and to assess the determinants of dietary diversity among women of reproductive age in two different agro-ecological zones of Rongai Sub-County in Kenya.

**Design:**

A cross-sectional study of 384 women aged 18–49 years was conducted. Agrobiodiversity was measured using the Shannon-Wiener index, species richness (count) and production diversity score. A 24-hour dietary recall was used to determine minimum dietary diversity for women (MDD-W) of reproductive age.

**Results:**

Although the level of agrobiodiversity was different between the low and high agro-ecological zones (using Shannon-Wiener index); the women’s dietary diversity was not different (*p* > 0.05) between low (3.78 ± 0.99) and high potential areas (3.84 ± 1.05). In multivariate logistic regression, there was no association (*p* > 0.05) between agrobiodiversity indicators and dietary diversity across the two agricultural zones. Factors influencing MDD-W in two agricultural zones were different. In low potential areas, woman’s education level positively determined dietary diversity, while in high potential areas household gender, woman’s education level, woman’s age and family size influenced MDD-W.

**Conclusion:**

The proportion of women who met minimum dietary diversity was low. Although agrobiodiversity was different in the two agro-ecological zones, women’s dietary diversity scores were similar. In low agricultural potential areas, only education level influenced women’s dietary diversity while household gender, education level, age and family size were the important determinants in high agricultural potential areas. Therefore, it is recommended that nutrition interventions focusing on lessening malnutrition and improving dietary quality should pay special attention to differences in agro-ecological zones to develop region-specific interventions instead of generalizing interventions.

## Popular scientific summary

- This study demonstrated that the diets of women were of poor quality despite the differences in agro-ecological zones and availability of food from the farm.
- Education level influenced women’s dietary diversity positively in low agricultural potential areas while household gender, education level, age and family size were important determinants in high potential areas.
- Nutrition interventions focusing on improving dietary quality of women should therefore pay special attention to developing region-specific interventions instead of generalizing interventions.

## 

Agrobiodiversity exists at numerous levels, from the various ecosystems in which people raise crops and livestock through different varieties and breeds of the species, to the genetic variability within each variety or breed (1). There is growing realization worldwide on the fundamental role of agrobiodiversity towards attaining food, nutrition security and sustainable agriculture (2–4). However, there is limited quantitative data available on the status of agrobiodiversity globally, though indications are that the loss of agrobiodiversity is occurring throughout the world at unprecedented rates (3). According to the FAO (3), it is estimated that about three-quarters of diversity found in agricultural crops has been lost over the last century, and this erosion continues. For instance, 90% of our food energy and protein comes from only 15 plant and 8 animal species, with alarming consequences for nutrition and food security (3).

The erosion of agrobiodiversity has coincided with reduction in dietary diversity (1, 4–5). Dietary diversity is the number of individual food items or food groups consumed over a given period of time (6). Dietary diversity is an essential element of diet quality; consuming a variety of foods across and within food groups is associated with adequate intake of essential nutrients and promotes good health (7, 8). However, limited accessibility to variety of foods to constitute diversified diets is a predominant problem among women of reproductive age in developing countries (9). Their diets consist mainly of starchy staples with few or no animal products, fresh fruits and vegetables (6, 10–11). Consumption of poor quality diets and general lack of access to wide food diversity has been acknowledged as among the major predisposing factors for maternal malnutrition (8, 12–13).

Globally, malnutrition is prevalent among women of reproductive age, where approximately 15% are underweight and 35% are overweight (14). In Kenya, analysis of body mass index shows that 9% of women aged 15–49 years are underweight while the proportion of overweight and obese women increased from 25% in 2008–2009 to 33% in 2015 (15). In addition, estimates from the Kenya Ministry of Public Health (16) show that micronutrient malnutrition is also predominant among women of reproductive age, where 48, 52 and 40% suffer from iron, zinc and vitamin A deficiency, respectively. Macro- and micronutrient deficiencies impose a large health burden in terms of lost productivity, increased susceptibility to diseases, impaired growth and development (17). Intake of high diverse diets has been associated with lower rates of malnutrition (18); hence increasing dietary diversity among women will be an important approach to improve their nutritional and health parameters. Moreover, women have increased nutrient needs during pregnancy and lactation, and when these requirements are not met women may suffer from malnutrition, which could negatively influence the developing foetus and the breastfeeding infant (19–21).

Agrobiodiversity is widely being perceived as a promising strategy to improve dietary quality and diversity (5, 22–24). Therefore, it is important to understand the relationship between agrobiodiversity and dietary diversity, as this could help in tackling the complex problem of malnutrition among women of reproductive age. However, it is not unanimously evident that high levels of agrobiodiversity will lead to better dietary diversity, with some studies supporting (5, 22–28) and others refuting (23, 29–30) the relationship. Thus, there arises a need to determine other factors that could significantly influence dietary diversity in different agro-ecological zones, as factors may vary from region to region. This identification of region-specific factors may help in developing nutrition-sensitive interventions that are particular to area characteristics instead of generalized interventions. Thus, the objective of this study was to document the status of agrobiodiversity and dietary diversity and to assess the determinants of dietary diversity among women of reproductive age in two different agro-ecological zones of Rongai Sub-County, Nakuru County, Kenya.

## Materials and methods

### Study area

The study was undertaken in Rongai Sub-County, Nakuru County (Kenya). The sub-county is divided into four divisions, namely: Ngata, Menengai, Kampi ya Moto and Solai. The subcounty lies in two different agro-ecological zones: the Upper Midland II zone (low potential) and Lower Highland II zone (high potential) (31). The two agro-ecological zones differ in amount of rainfall received. The low potential areas receive an average rainfall of between 760 and 900 mm and high potential areas receive an average rainfall of between 900 and 1,270 mm (32). Moreover, the soil types vary in the two agro-ecological zones. In low potential areas the soils are alluvial and lacustrine deposit. These are shallow soils developed from sediments of volcanic ashes and have low to moderate fertility. In high potential areas the soils are mainly latosolic and planosolic soils; these are highly developed, textured top soils, well drained and with high fertility. The common agricultural activity in low potential is livestock keeping, and the major crops grown are maize, sorghum, millet and cassava; while in high potential areas the common agricultural activities are crop and dairy farming with the major crops being wheat, maize, beans, sunflowers, vegetables, peas and potatoes (32).

### Survey design, population and sampling

A cross-sectional survey was conducted from March to April 2016, to assess the socio-demographic characteristics, agrobiodiversity and dietary diversity of 384 women of reproductive age in Rongai Sub-County. This study was part of a larger project determining the relationship between agrobiodiversity and the dietary diversity of women and young children (12–23 months) in Rongai Sub-County in Kenya. This study focused specifically on the relationship between agrobiodiversity and dietary diversity and factors influencing the dietary diversity of women of reproductive age in Rongai Sub-County.a

The sampling frame were all women of reproductive age from 18 to 49 years in the Kampi ya Moto and Menengai divisions of Rongai Sub-County, who were smallholder farmers and had lived in Rongai Sub-County for at least 1 year prior to the study. The sampling frame was obtained from the Ministry of Agriculture, Nakuru County population records.

The study sample size was determined using Fischer’s formula (33), $\text{n=}\frac{{\text{z}}^{\text{2}}\text{pq}}{{\text{e}}^{\text{2}}}$ ÷ where (Z), the normal deviation, was 1.96 set at 95% confidence interval, and *P* was 39%, the estimated prevalence of malnourished women in the study area (15). An attrition rate of 10% was factored into the sample size determination, and the sample size was adjusted to 400. The number of participants was then selected from each agro-ecological zones using the probability proportional to size method; in low potential areas, 41% (*n* = 166), and in high potential areas, 59% (*n* = 234). Some variables such as crop species cultivated and livestock reared by households were missing from 16 respondents; hence the data was not included in the analysis.

Two multistage cluster sampling procedures were adopted to obtain an appropriate sample for the study. In the first sampling stage the two divisions were purposively selected to capture two agro-ecological zones: a low potential area (Kampi ya Moto Division) and a high potential area (Menengai Division). Four sublocations were then selected randomly from each division. All smallholder farming households with women of reproductive age from each sublocation were listed, and probability random sampling proportion to size was used to select the study participants. Random sampling was carried out in households that had more than one woman of reproductive age, in order to select one woman to participate in the study. Before any data was collected, informed written consent was obtained from each participant. This was done after the researcher explained the purpose, risk and expected outcomes of the study to the respondents. Ethical approval to conduct the research was granted by the National Council of Science and Technology, Kenya.

## Data collection

### Study variables

The dependent variable for this study was minimum dietary diversity for women (MDD-W) of reproductive age (34) and the agrobiodiversity indicators (Shannon-Wiener index, species richness [count] and production diversity score) were the independent variables. Other independent variables in the model were household gender, household income, woman’s education level, woman’s age, household size, household wealth index and farm size.

### Socio-demographic characteristics

A structured household questionnaire was used to gather information on socio-demographic and household characteristics such as valuable assets including electricity, mobile phone, television, type of roofing material, type of fuel and type of toilet facilities. This information was used to generate a household wealth index following the principal component analysis technique (15). The household wealth index was used as a proxy for household’s socio-economic status. A brief description of the main independent and dependent variable is given in the following.

### Measurements of agrobiodiversity

The structured household questionnaire was also used to gather information on food crops and animal species diversity on household farms. All on-farm species present were classified into five categories: 1) cereals, tubers and roots; 2) legumes and nuts; 3) fruits; 4) vegetables; and 5) domesticated animals. Agrobiodiversity was measured using the Shannon-Wiener index for crop species and a species richness (count) combined for crops and animals (30–35). The Shannon-Wiener index is a diversity index used to reflect species richness (count) and evenness (abundance) (35, 36). A definitive cut-off point does not bind the Shannon-Weiner index score; thus an increase in the score reflects greater diversity in a household farm (35, 36).

To incorporate both plants and livestock in a single farm diversity measure, a combined crop and livestock count was computed (22, 30, 37) by summing up the numbers of different food plant species and livestock that were cultivated or reared by each household. The single farm diversity measure was termed ‘species richness’ or ‘count’. The species richness or count indicator does not discriminate crops based on how much land they occupy; rather it considers trait differences as the most important element for diversity. A definitive cut-off point does not bind the count; the higher the score, the more diverse household farm is (36). However, a species count does not necessarily reflect diversity from a dietary point of view (38). Hence, to better account for the dietary perspective, a production diversity score was generated. The production diversity score is defined as the number of food groups produced on a farm (38, 39). To construct the production diversity score, the scoring used to generate the women’s dietary diversity score was adapted by considering the 10 recommended food groups (34). If a farm had sorghum, maize and millet (all cereals), this was counted as ‘1’, and those farms that didn’t cultivate any cereals were assigned ‘0’. The same was done for the other food groups, and the scores from all food groups were summed up to obtain the production diversity score (38, 39).

### Measurement of dietary diversity

A semi-quantitative 24-h recall questionnaire was used to gather information on all foods and beverages consumed by each participant in the previous 24 h (40). This information was then used to generate the MDD-W of reproductive age scores by aggregating the foods consumed into 10 recommended food groups (34). The 10 food groups include: 1) starchy staples; 2) pulses – beans, peas and lentils; 3) nuts and seeds; 4) dairy products; 5) meat, poultry and fish; 6) eggs; 7) dark green leafy vegetables; 8) vitamin A rich fruits and vegetables; 9) other vegetables; and 10) other fruits.

### Data analysis

Data was coded and analysis performed using IBM SPSS complex sample version 20 (module). Data analysis took into account the complex design of multistage cluster sampling. This was done to make statistically valid population inferences and to compute standard errors from the sample data. Weighted analysis was performed by applying sample weight to each cluster to account for the difference in population size. Means and percentages were used to describe the data. A chi-square (χ^2^) test was used to compare categorical variables such as household gender, woman’s marital status, education level and dietary diversity in the two agro-ecological zones.

Independent samples *t*-tests were used to compare two means such as age, household size, Shannon-Wiener index, species richness (count) and the production diversity score from the two agro-ecological zones. Two independent bivariate and multivariate analyses were carried out to identify different determinants of dietary diversity across the two ecological zones. The independent variables with a *p*-value less than 0.2 with the dependent variable were fitted into a multivariate logistic regression model to identify their independent effect on dietary diversity. The dependent variable was MDD-W, with two categories: low and high dietary diversity. The categories were based on the minimum threshold of dietary diversity among women of reproductive age, which is consumption of 5 or more out of the 10 recommended food groups. Women who consumed five or more food groups were categorized as having high dietary diversity, while those consuming less than five food groups were categorized as having low dietary diversity (41). Independent variables included in the multivariate analysis were household head’s gender, household level of income, household wealth index, household size, woman’s education level and age, cultivated farm size and agrobiodiversity indicators. Multicollinearity was investigated using the variance inflation factor (VIF). The VIF factor ranged from 2.480 to 4.335, which were below the suggested cut-offs (>5), above which collinearity is considered a problem (42). The direction and strength of association between the dependent and independent variables were assessed using the regression coefficient and adjusted odds ratio (AOR) with 95% confidence interval. The *p*-values <0.05 were considered statistically significant.

## Ethical considerations

Ethical clearance was obtained from Egerton Research Ethics Committee. A research permit was obtained from the National Council of Science and Technology, Kenya. Permission was also obtained from relevant authorities at subcounty, location and sublocation level. The researcher clarified the purpose of the study to the respondents and the questionnaires were administered upon obtaining informed consent.

## Results

### Socio-demographic and economic characteristics of the study population

From the 384 households of the study, the majority were male headed, with 82% in low potential areas and 81% in high potential areas. Most of the women were married (73 and 80%) in low and high potential areas, respectively (Table 1). Forty per cent of the women had attained primary-level education across the two agro-ecological zones. There was a significant (*p* < 0.05) difference in wealth distribution in the two agro-ecological zones. Low potential areas had a higher proportion of households in the poorest category (70%) compared to high potential zones (56%). Few households were in the rich category; with a higher proportion from high potential areas (5%) compared to low potential areas (1%). On average, the total number of household members in the low potential areas (5.78 ± 2.54) was significantly (*p* < 0.05) higher than in high potential areas (4.98 ± 2.35). In general, each household had approximately five members (5.78 ± 2.54) in low and (4.98 ± 2.35) in high potential areas and an average of about three children across the two agro-ecological zones (Table 1).

**Table 1:** Socio-demographic and socio-economic characteristics of women of reproductive age in low and high agricultural potential areas of Rongai Sub-County

Characteristic	Agro-ecological zones				*P*
	Low potential area (*n* = 159)		High potential area (*n* = 225)		
	%	*n*	%	*n*	
HH head sex				
Male	82	131	81	183
Female	18	28	19	42	0.792
Women’s characteristics Marital status^a^				
Married	72	115	80	181
Single	25	40	17	39	0.166
Widowed	1	2	3	6
Separated	1	2	0.4	1
Religion^a^				
Muslim	10	16	3	7
Christian	90	143	97	218	0.005*
Ethnicity^a^				
Kalenjin	74	117	56	125
Kikuyu	12	19	26	58	0.001**
Other^b^	15	23	19	42
Education^a^				
None	10	16	12	26.
Primary	40	64	44	100	0.400
Secondary	37	59	29	65
Tertiary	13	20	15	34
Wealth index categories				
Poorest	70	108	56	125
Poor	28	43	36	36	0.018*
Middle	2	3	3	38
Rich	1	1	5	11
	**Means±SD**		**Means±SD**		***P***
Age in years^c^	28.44 ± 8.51		29.55 ± 9.59		*P* = 0.244
HH size^c^	5.78 ± 2.54		4.98 ±2.35		*P* = 0.002*
Average number of children^c^	2.81 ± 2.02		2.79 ± 1.93		*P* = 0.904

HH, household,

a characteristic of the women of reproductive age;

b other ethnic groups include Kisii, Turkana, Luhya, Meru and Mijikenda;

* *p* < 0.05,

** *p* < 0.01 significant by χ^2^ test;

c data are mean ± standard deviations;

* *p* < 0.05 significant using independent samples *t*-test.

### Agrobiodiversity status in the two agro-ecological zones

The Shannon index for all species (edible and non-edible crops) was significantly (*p* < 0.05) lower among households in low potential areas (0.96 ± 0.48) compared to those in high potential areas (1.10 ± 0.43). The mean diversity of edible species (crops) was higher in high potential areas (1.08 ± 0.41) as compared to low potential areas (0.93 ± 0.40) (*p* < 0.05).

Furthermore, the measurement of agrobiodiversity using the species count (richness) indicator showed higher (*p* < 0.05) diversity of cereals, tubers and roots in high potential areas (1.75 ± 0.83) compared to low potential areas (1.49 ± 0.86), while diversity of legumes, nuts and domesticated animals was higher in low potential areas (Table 2). There was no difference (*p* < 0.05) in the diversity of fruits, vegetables and species richness. However, total crop count was higher (*p* < 0.05) in high potential areas (4.53 ± 2.22) than low potential areas (3.95 ± 2.53).

**Table 2:** Species richness status in low and high agricultural potential areas of Rongai Sub-County

Categories	Agro-ecological zones		*p*
	Low potential (*n* = 159)	High potential (*n* = 225)	
Cereals, tubers and roots	1.49 ± 0.86	1.75 ± 0.83	0.005*
Legumes and nuts	1.05 ± 0.26	1.00 ± 0.00	0.019*
Fruits	2.18 ± 1.11	1.76 ± 1.14	0.075
Vegetables	2.24 ± 1.13	2.28 ± 1.25	0.799
Domesticated animals	2.29 ± 1.01	1.93 ± 0.91	0.001**
Total crop count	3.95 ± 2.53	4.53 ± 2.22	0.018*
Species count (richness)	5.77 ± 6.21	6.17 ± 2.78	0.220

HH, household;

* *p* < 0.05,

** *p* < 0.01 significant using independent samples *t*-test, total crop count^a^, an aggregate of all crops (cereals, legumes and nuts, fruits and vegetables).

### Frequency of food groups produced by farm households

The proportion of farms producing the 10 food groups recommended by the FAO (34) in the dietary diversity score (DDS) in Rongai is shown in Table 3. A high percentage of farm households produced starchy staples with high potential areas producing more starchy staples 93% compared to low potential areas 84%. Pulses were cultivated by more than 80% of households in both low and high potential areas while vitamin A rich foods, other fruits, nuts and seeds were least produced across the two agro-ecological zones. More households (31%) in high potential areas grew other vegetables compared to low potential areas 13%. More than half of households produced dairy products with more farm households in low potential areas (62%) than in high potential areas (53%). The average production diversity score (number of DDS food group produced per farm) was approximately 5 food groups per farm households, with high potential areas producing more food groups (5.25 ± 1.99) compared to low potential areas (4.94 ± 2.26).

**Table 3:** Proportion of households producing foods from the different food groups in low and high agricultural potential areas of Rongai Sub-County

Food groups produced	Agro-ecological zones				*p*
	Low potential (*n* = 159)		High potential (n = 225)		
	%	*n*	%	*n*	
Starchy staples	84	132	93	210	0.004*
Pulses	83	130	85	192	0.504
Nuts and seeds	1	1	0	0	-b
Vitamin A–rich fruits and vegetables	29	45	25	56	0.411
Dark green leafy vegetables	50	78	60	134	0.056
Other vegetables	13	20	31	69	0.001*
Other fruits	21	33	24	54	0.494
Dairy products	62	96	53	120	0.130
Eggs	68	106	68	154	0.848
Meat, poultry and fish	85	134	86	193	0.907
Production diversity score^a^	4.94 ± 2.258		5.25 ± 1.986		0.125

* *p* < 0.05 significant by χ^2^ test,

a data are mean ± standard deviations;

* *p* < 0.05 significant using independent samples *t*-test,

b the value could not be estimated because of the small samples.

### Dietary diversity of women in the two agro-ecological zones

Overall, the DDS of women was 3.78 ± 0.99, with no difference (*p* > 0.05) between women residing in low (3.78 ± 0.99) and high potential areas (3.84 ± 1.05). In addition, over 75% of the women consumed foods from fewer than five food groups, thus not meeting the MDD threshold (consumption of five or more food groups). However, a higher proportion of women from high potential areas met minimum dietary diversity (22% – consumed five or more food groups) compared to 16% from the low potential areas as shown in Table 4.

**Table 4:** Proportion of women of reproductive age consuming items from 10 foods groups over the previous 24 h in low and high agricultural potential areas of Rongai Sub-County

	Agro-ecological zones				
	Low potential (*n* = 159)		High potential (*n* = 225)		*p*
Food groups^a^	%	*n*	%	*n*
Starchy staples	99	158	100	225	0.234
Pulses	46	73	40	90	0.248
Nuts and seeds	1	1	0	0	-b
Dairy products	15	24	28	62	0.004*
Meat, poultry and fish	10	16	9	21	0.811
Eggs	6	9	6	14	0.819
Dark green leafy vegetables	84	133	83	186	0.801
Vitamin A–rich fruits and vegetables	13	20	15	34	0.482
Other vegetables Other fruits	91	144	93	210	0.320
Dietary diversity categories	8	12	8	19	0.774
Low dietary diversity ≤5 food groups	84	133	78	175	0.155
High dietary diversity ≥5 food groups	16	26	22	50

* *p* < 0.05 significant by χ^2^ test,

a responses were dichotomized to ‘yes’ or ‘no’ – the data presented is for ‘yes’,

b the value could not be estimated because of the small sample.

### Proportion of food groups consumed by women in the two agro-ecological zones

The frequency of food groups consumed by women is shown in Table 4. A higher percentage of women consumed starchy staples, with 99% and 100% in low and high potential areas, respectively. Nuts and seeds, meat, poultry, fish and eggs were the least consumed across the two agro-ecological zones. Vegetables formed an integral part of the main meals, with 84% and 83% of women consuming dark-green leafy vegetables in low and high potential areas, respectively. Other vegetables were consumed by more than 90% across the two agro-ecological zones. Vitamin A–rich fruits and vegetables were consumed by less than 20%, which was low in the two agro-ecological zones. More women from high potential areas (28%) consumed dairy products compared to those from low potential areas (15%).

**Fig. 1:**
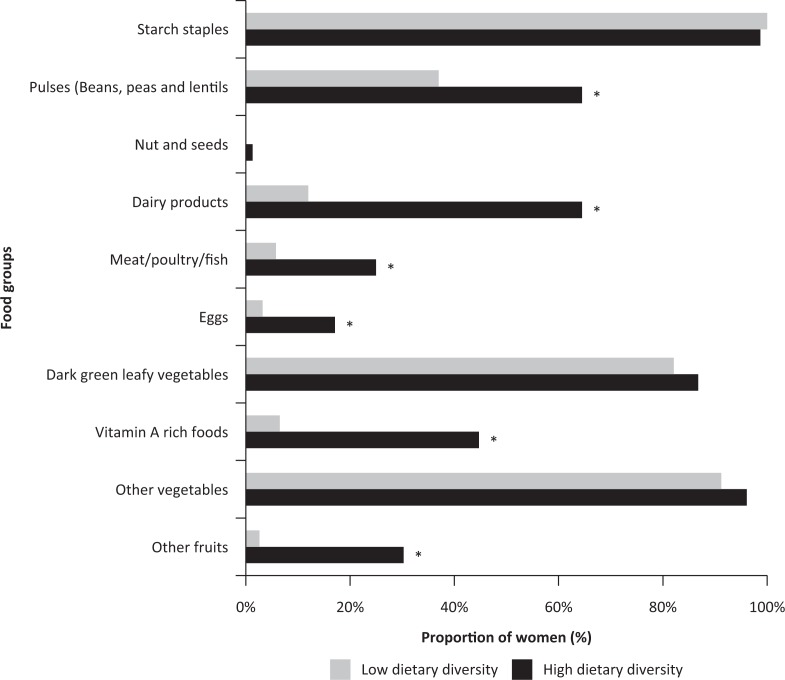
Proportion of women of reproductive age with low versus high dietary diversity score consuming each of the 10 food groups over 24 h. ‘High’ represents those consuming 5 out of 10 food groups (MDD-W) or more [10], whereas ‘low’ represents those consuming 4 food groups or less. *p < 0.05 significant by χ^2^ test. MDD-W, minimum dietary diversity for women.

Further, the proportion of women consuming foods from various foods groups differed between the two dietary diversity categories (those with low and high DDS scores). Starchy staples, dark green leafy vegetables and other vegetables were the most frequently consumed food groups by women in both categories (Fig. 1). More women with high DDS consumed pulses (65%), dairy products (65%) and vitamin A–rich foods (45%) compared to women with low DDS (37, 12, and 7%, respectively). Nuts and seeds (0%), eggs (3%), other fruits (3%) and meat/poultry/fish (6%) were the least consumed food groups by women with low DDS.

### Determinants of dietary diversity of women in the two agro-ecological zones

The factors that influence dietary diversity were found to be different in the two agro-ecological zones (Table 5). In low agricultural potential areas, only woman’s education level positively (*p* < 0.05) influenced dietary diversity. Women with a high education level were 3.65 times more likely (AOR = 3.65, 95% confidence interval [CI] [1.21–10.99]) to have high dietary diversity than those with low education. The household gender, woman’s education level, woman’s age and family size influenced dietary diversity in high potential areas. Women from male-headed households were 4.15 times more likely (AOR = 4.15 [1.16–14.86], *p* < 0.05) to have high dietary diversity compared to those from female-headed households. Women with a high education level were 5.32 times (AOR = 5.32, 95% CI [2.27–12.46], *p* < 0.05) more likely to have high dietary diversity than those with low education. In addition, older women were most likely to have a diverse diet (AOR = 1.13, 95% CI [1.07–1.18], *p* < 0.01); however, larger household size negatively influenced dietary diversity (AOR = 0.77; CI [0.62–0.95], *p* < 0.05]. There was no association (*p* > 0.05) between dietary diversity and household income, wealth index, Shannon index, species richness and production score across the two agricultural zones.

**Table 5:** Multivariable analysis of the determinants of dietary diversity score for women of reproductive age in low and high agricultural potential areas of Rongai Sub-County

	Agro-ecological zones			
	Low potential zone (*n* =159)		High potential zone (*n* = 225)	
Factors	UOR (95% CI)#	AOR (95% CI)#	UOR(95% CI)#	AOR (95% CI)#
Household gender			
Female (reference)		1		1
Male	3.30 (0.94–11.61)	2.85 (0.68–11.88)	1.59 (0.66–3.83)	4.15 (1.16–14.86)*
Household income				
Low income (reference)		1		1
High income	2.066 (0.88–4.87)	1.35 (0.46–3.95)	2.15 (1.11–4.16)*	1.50 (0.58–3.89)
Woman’s education level				
Low (reference)		1		1
High	4.18 (1.58–11.08)	3.65 (1.21–10.99)*	5.18 (2.57–10.49)*	5.32 (2.27–12.46)*
Woman’s age	1.02 (0.98–1.08)	1.05 (0.99–1.12)	1.09 (1.05–1.14)*	1.13 (1.07–1.18)*
Household size	0.94 (0.79–1.10)*	0.88 (0.71–1.09)	0.89 (0.77–1.04)*	0.77 (0.62–0.95)*
Household wealth index	1.79 (1.12–2.89)	1.32 (0.71–2.46)	1.38 (1.06–1.80)	0.86 (0.55–1.33)
Farm size (acres)	1.20 (0.91–1.58)	0.92 (0.58–1.45)	1.25 (1.01–1.55)	1.30 (0.97–1.75)
Shannon index for edible crops	1.17 (0.55–2.48)	0.37 (0.06–2.17)	1.85 (0.82–4.19)	1.14 (0.31–4.16)
Species richness (count)	1.02 (0.90–1.15)	0.95 (0.81–1.11)	1.16 (1.04–1.30)	0.93 (0.82–1.06)
Production diversity score	1.11 (0.90–1.37)	1.56 (0.87–2.80)	1.28 (1.04–1.56)	1.30 (0.89–1.89)

* p < 0.05, p < 0.01 significant using binary logistic regression; UOR, unadjusted odds ratios; CI, confidence interval, AOR, adjusted odds ratio; adjusted for household gender, household income, agro-ecological zones, woman’s education level, woman’s age, family size, household wealth index, cultivated farm size in acres, Shannon index for edible crops, total agrobiodiversity count and production diversity score.

## Discussion

This study demonstrated that the determinant of a quality diet in low agricultural potential areas was woman’s education level while in high agricultural potential areas, important determinants were household gender, woman’s education level, woman’s age and family size. The study also shows that the proportion of women who met minimum dietary diversity was low and not different between low and high potential areas despite the differences in agrobiodiversity. A possible explanation for this finding may be a low level of knowledge or lack of it on the utilization of local agrobiodiversity to improve diets. Lack of knowledge on the locally available nutrient-rich foods (agrobiodiversity), and how best to utilize them in the diet, has resulted in these foods being underutilized and neglected (43).

This study showed the existence of low dietary diversity among women of reproductive age in Rongai Sub-County indicating poor dietary quality. Women in resource-poor settings are at risk of inadequate dietary intakes (9, 44). Poor dietary diversity is well recognized as a critical factor for maternal undernutrition. Maternal undernutrition is a major predisposing factor for morbidity and mortality in women, notably caused by inadequate food intake and poor diet quality (15, 45). Importantly, women who are undernourished are at higher risk of having pregnancy complications and labour problems, and they recover more slowly from illnesses (15). Maternal undernutrition also contributes to foetal growth restriction, which increases the risk of neonatal deaths, and survivors tend to be at a higher risk of stunting (46). Hence, this makes the need for nutrition-sensitive interventions to diversify women’s diets paramount. The key features that make these interventions of utmost importance is that they address crucial underlying determinants of nutrition; they are often implemented on a large scale and can be effective at reaching poor populations that have high malnutrition rates (47).

In low agricultural potential areas, only woman’s education level positively influenced dietary diversity while the household gender, woman’s education level, woman’s age and family size influenced dietary diversity in high potential areas. In the two zones, educated women were more likely to have high dietary diversity. Note that educated women assign a significantly more substantial proportion of their household food budget to nutritious foods (48, 49). This is mainly because educated women tend to have greater awareness and understanding of nutritional health benefits (45); moreover, an educated woman is an empowered woman. Women’s education is recognized as a critical factor for women’s empowerment. This enables them to gain greater access and control over financial and knowledge resources to improve their lives (50). Studies have shown the important linkages between women’s empowerment dimensions and nutritional outcomes. Improvements in various empowerment indicators have been associated with enhancement in maternal and child nutrition; conversely, women’s disempowerment is associated with poor child and maternal health and nutrition outcomes (51).

In high agricultural potential areas, women’s dietary diversity was also influenced by the gender of the household head, with those headed by men having higher dietary diversity. Similar findings were reported in a study in Ethiopia (52) that assessed nutritional parameters in relation to gender differences. In that study, dietary intake was disaggregated by household type, and it was demonstrated that the nutrient intake in male-headed households was relatively better than in female-headed households, though the difference was not statistically significant. Such manifestations are usual as male-headed households have the advantage of more sources of income instead of one, especially when both partners are involved in revenue-generating activities. The combined income of both spouses probably offers them better opportunities to access a variety of different food products, thus increasing their dietary diversity. The increased household size was another factor that negatively influenced women’s dietary diversity in high potential areas. This could partly be explained by the fact that as the number of family members increases, the intra-household food distribution is affected and food may become more limited, which in turn would limit access to different food groups.

Although the level of agrobiodiversity was different between the low and high agro-ecological zones, the dietary diversity of the women remained the same. This could allude to the fact that availability of food from the farm does not always translate to better dietary diversity. These findings concur with other studies (29, 30, 53–55), which demonstrated that a rich biodiverse environment does not contribute substantially to better diets among rural women. However, other reports (22, 26, 38, 55, 56) have shown a positive relationship between measures of agrobiodiversity and dietary diversity.

A positive association between farm production diversity and dietary diversity should be plausible. As households practising small-scale farming tend to consume a considerable share of what they produce, agrobiodiversity should then directly translate into consumption diversity and consequently improve dietary quality through this production pathway (37). However, in this study, there was no significant association between agrobiodiversity measures and dietary diversity of women in the two agro-ecological zones. This lack of connection could be attributed to market diversity, which is a major mediating factor in the relationship between agrobiodiversity and dietary diversity (56, 57). Taking market diversity into account, the relationship between agrobiodiversity and dietary diversity becomes more complicated (57). Instead of producing foods from all food groups at home, farm households buy food from the market, which can contribute to improving dietary diversity. However, markets can worsen dietary diversity if the households sell the nutritious food products to obtain income with which to cater for family needs such as school fees. The common practice in both wealthy and poor households is selling of farm produce, especially immediately after the main harvest seasons (30, 58). A study by Sibhatu et al. (37) documented a negative significant interaction between market diversity and agrobiodiversity and confirmed that market participation by the households could reduce the role of agrobiodiversity in improving dietary diversity.

The production diversity score also had no significant relationship with dietary diversity. Sibhatu et al. (37) found out that when using production diversity scores instead of a simple species count, the effect on dietary quality got smaller; in many cases, it turned insignificant. This intriguing finding was in line with the findings of the current study. The production diversity score measures the number of different food groups produced on a farm, so one could have expected the effect of production diversity on the number of food groups consumed in the farm household to be stronger. The fact that this is not the case reveals that the subsistence pathway is not the only mechanism underlying the production–consumption relationship (37). Market diversity through purchase or sale of diverse food products seems to be another critical factor that could contribute to improving dietary quality. Therefore, further research is needed to elucidate these dynamics and to comprehend the region-specific factors that may influence the role of markets in moderating the relationship between agrobiodiversity and dietary diversity.

The linkages between agrobiodiversity, market diversity and dietary diversity are complex (37, 57). For instance, in some cases, agrobiodiversity in the farm household may be high and a wide range of food crops available in the markets, but this does not automatically translate to higher DDS. Factors such as intrahousehold resource and food allocation may come into the interplay further complicating this relationship. Even when the food is available in the farms or markets, the intrahousehold allocation of food may disfavour women’s access to nutritious foods because of cultural beliefs, economic constraints and low decision-making. For example, in many regions of South Asia, women find themselves in subordinate positions to men; they tend to eat the least, or to eat leftovers after other family members have eaten (59–60). Moreover, women are largely excluded from making decisions, have limited access to and control of resources and are restricted from mobility by their husbands and sometimes by in-laws (60–62). This compromised access leads women to make suboptimal decisions with regard to food choices, which may subsequently cause poor dietary intake.

The determinants of dietary intake are complex and are dependent on a wide range of diverse and interconnected factors. The UNICEF conceptual framework of determinants of malnutrition gives a detailed overview of the factors that influence dietary intake (63). Hence, the current study has brought out some of the determinants of dietary diversity, which is a component of dietary intake.

## Conclusion

From a ‘nutrition-sensitive’ policy perspective, our findings draw attention to different factors influencing dietary diversity in the two agricultural zones. Household gender (male), higher woman’s education level, older age and smaller families are essential determinants in improving the dietary diversity of women in rural areas. The following four policy inferences are suggested for successful implementation of nutrition interventions in Rongai Sub-County and other similar agricultural areas: Firstly, nutrition interventions should not be too general and should take specific factors and conditions to agro-ecological environments into consideration. Secondly, family planning needs to be intensified because large household sizes impact on women’s dietary diversity. Thirdly, education for women should be emphasized to promote women empowerment so as to enable them to gain greater access and control over financial and knowledge resources to improve their lives and diets. Although there were differences in agrobiodiversity characteristics between the two agricultural potential areas, this did not translate into differences in diet diversity for women of reproductive age. Lastly, there is a need for behaviour change communication to ensure that locally available agrobiodiversity is utilized to improve the diet quality of women of reproductive age.
